# Sublethal exposure to alpha radiation (^223^Ra dichloride) enhances various carcinomas’ sensitivity to lysis by antigen-specific cytotoxic T lymphocytes through calreticulin-mediated immunogenic modulation

**DOI:** 10.18632/oncotarget.13520

**Published:** 2016-11-17

**Authors:** Anthony S. Malamas, Sofia R. Gameiro, Karin M. Knudson, James W. Hodge

**Affiliations:** ^1^ Laboratory of Tumor Immunology and Biology, Center for Cancer Research, National Cancer Institute, National Institutes of Health, Bethesda, MD, USA

**Keywords:** radium-223, alpha radiation, CTL-mediated lysis, immunogenic modulation, calreticulin

## Abstract

Radium-223 dichloride (Xofigo^®^; ^223^Ra) is an alpha-emitting radiopharmaceutical FDA-approved for the treatment of bone metastases in patients with advanced castration-resistant prostate cancer. It is also being examined clinically in patients with breast and lung carcinoma and patients with multiple myeloma. As with other forms of radiation, the aim of ^223^Ra is to reduce tumor burden by directly killing tumor cells. External beam (photon) and proton radiation have been shown to augment tumor sensitivity to antigen-specific CD8^+^ cytotoxic T lymphocytes (CTLs). However, little is known about whether treatment with ^223^Ra can also induce such immunogenic modulation in tumor cells that survive irradiation. We examined these effects *in vitro* by exposing human prostate, breast, and lung carcinoma cells to sublethal doses of ^223^Ra. ^223^Ra significantly enhanced T cell-mediated lysis of each tumor type by CD8^+^ CTLs specific for MUC-1, brachyury, and CEA tumor antigens. Immunofluorescence analysis revealed that the increase in CTL killing was accompanied by augmented protein expression of MHC-I and calreticulin in each tumor type, molecules that are essential for efficient antigen presentation. Enhanced tumor-cell lysis was facilitated by calreticulin surface translocation following ^223^Ra exposure. The phenotypic changes observed after treatment appear to be mediated by induction of the endoplasmic reticulum stress response pathway. By rendering tumor cells more susceptible to T cell-mediated lysis, ^223^Ra may potentially be effective in combination with various immunotherapies, particularly cancer vaccines that are designed to generate and expand patients’ endogenous antigen-specific T-cell populations against specific tumor antigens.

## INTRODUCTION

Prostate cancer is one of the most prevalent malignancies among men worldwide. Although the 5-year survival rate is > 90% in patients with localized disease, the rate is < 30% in patients who develop distant metastases, typically after the primary tumor becomes insensitive to androgen-deprivation therapy [1]. Castration-resistant prostate cancer (CRPC) has a high tendency to metastasize to bone, leading to various skeletal-related events, decreased bone strength, spinal cord compression fractures, debilitating pain, and bone marrow failure [2, 3]. Treatment of metastatic CRPC (mCRPC) includes the use of bisphosphonates and the anti-RANKL antibody denosumab, which act on the bone environment and reduce the incidence of skeletal-related events [4–6]. However, both treatments fail to prevent metastasis and do not improve either progression-free or overall survival [7].

Radiotherapy is another option for treating mCRPC. Targeted therapeutic radioisotopes are preferred over external beam radiation therapy (EBRT) for patients with diffuse, widespread bone involvement because they spare normal tissue from unnecessary irradiation [8, 9]. Radium-223 dichloride (Xofigo^®^; ^223^Ra) is the first of a new class of alpha-emitting radiopharmaceuticals to be approved by the U.S. Food and Drug Administration (FDA) for the treatment of bone metastases in mCRPC. Due to its chemical similarity to calcium, ^223^Ra forms complexes with the bone mineral hydroxyapatite at areas of increased osteoblastic activity, such as bone metastases [10–13], and has demonstrated an overall survival benefit in patients receiving intravenous treatment. The beta-emitting radionuclides previously approved by the FDA, strontium-89 (^89^Sr) and samarium-153 (^153^Sm), provide palliation for patients with multifocal bone metastases, but have failed to extend overall survival when given as monotherapies [9, 10].

The primary advantage of treating metastases with alpha-emitting rather than beta-emitting radionuclides is the potential to deliver a greater dose of radiation in a highly localized manner [14, 15]. Alpha particles are heavily charged, composed as they are of a helium nucleus with 2 positive charges, while beta particles are much smaller and take the form of either electrons or positrons. Furthermore, the path lengths of ^89^Sr and ^153^Sm are millimeters in both soft tissue and bone, while the range of alpha particles emitted from ^223^Ra is < 100 μm, or approximately 10 cell diameters in length [10, 15]. This short range of activity limits damage to surrounding normal tissue, leading to improved safety profiles compared to beta-emitting therapies. In particular, ^223^Ra delivered to bone surfaces has been reported to spare the bone marrow compartment and minimize the myelosuppressive effects observed with beta emitters [14, 16]. On the other hand, the linear energy transfer of alpha particles is about 100–1000 times greater than the average linear energy transfer of beta particles [13]. This ultimately leads to a relatively higher frequency of complex DNA double-strand breaks that are more difficult to repair than those created by beta emitters [9, 13, 14]. Still, phase III clinical studies in mCRPC have demonstrated that ^223^Ra is able to improve patient survival with limited toxicity, while having positive effects on changes in serum alkaline phosphatase and prostate-specific antigen (PSA) biomarker levels. In addition, the improvement in survival appears to be independent of prior exposure to both docetaxel and bisphosphonates [17].

Although the cytotoxic effects of ^223^Ra are well studied, it is currently unknown if this alpha emitter can act synergistically with immunotherapy strategies to enhance the killing of tumor cells that survive this treatment modality. Some tumor cells survive sublethal radiation due to dosing constraints dictated by the need to minimize toxicity to the patient, which often translates into dose heterogeneity within a given tumor mass. In addition to initiating a diverse immune response through antigen cascade, radiation therapy can also alter the phenotype of the surviving tumor cells in ways that render them more susceptible to cytotoxic T lymphocyte (CTL)-mediated attack. This process, known as immunogenic modulation, largely involves the regulation of molecules involved in antigen presentation, which can be critical for effective T-cell recognition [18–21]. Such events have been extensively characterized in a variety of human carcinoma cell lines following EBRT and proton radiation therapy *in vitro* [22]. We have also demonstrated clinically the immunomodulatory effects of EBRT, as it elicits a greater tumor antigen-specific CD8 T-cell response and a consequent reduction in tumor burden in combination with vaccine than with either modality alone.

In this study we explored the ability of ^223^Ra to induce immunogenic modulation and enhance CTL-mediated lysis of human prostate, breast, and lung carcinoma cell lines, each harboring a different p53, triple-negative, or K-Ras mutational status, respectively. While ^223^Ra is already FDA-approved for the treatment of mCRPC, it may also have clinical benefit against breast and lung cancers, as they also frequently metastasize to bone [23, 24].

## RESULTS

### Increasing doses of ^223^Ra inhibited cell proliferation with minimal effect on viability

The goal of this study was to analyze the immunomodulatory effects of alpha-emitting ^223^Ra on a variety of tumor types, and to determine whether those phenotypic changes enhanced CTL-mediated lysis of the surviving tumor-cell population. Therefore, to establish a nonlethal dose for subsequent experiments, we needs to understand how ^223^Ra affects both cell viability and proliferation over a range of doses *in vitro*. Over a 96-hour period, we exposed prostate (LNCaP and PC3), breast (MDA-MB-231 and ZR75-1), and lung (H441 and H1703) carcinoma cells (1 × 10^6^ cells) to 0, 2, 4, 10, and 40 Gy of ^223^Ra, a range that includes and exceeds clinically relevant doses. We strategically chose 2 prostate, breast, and lung tumor lines that were genotypically distinct based on their p53, triple-negative, or K-Ras mutational status, respectively (Table 1).

**Table 1:** Human carcinoma cell lines of diverse origin and phenotype treated with ^223^Ra alpha radiation

Tumor Class	Cell Line	Notable Characteristic
Breast	MDA-MB-231	Triple Negative (ER–/PR–/Her2neu–)
	ZR75-1	Triple Positive (ER+/PR+/Her2neu+)
Prostate	LNCaP	P53 Wild type
	PC3	P53 Null
Lung	H1703	Ras Wild Type
	H441	Ras Mutant (G12V)

**Figure 1:**
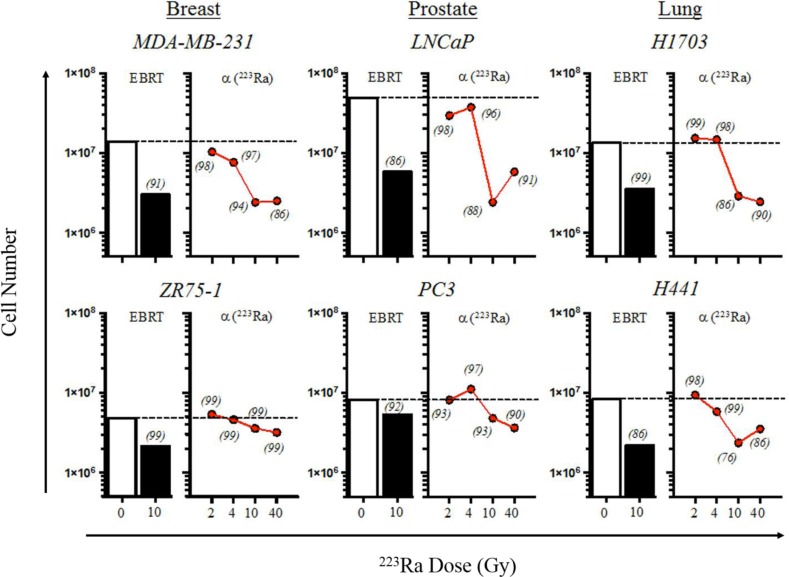
^223^Ra alpha radiation inhibits tumor proliferation, with minimal effects on cell viability Human prostate, breast, and lung carcinoma cells were mock-irradiated (0 Gy, open bars) or treated with either 10 Gy of photon radiation (EBRT, closed bars) or 2–40 Gy of ^223^Ra (red circles). EBRT-treated cells were irradiated and then cultured for an additional 96 h. Alpha-irradiated cells were cultured with an appropriate amount of ^223^Ra so that the total cumulative dose was 2, 4, 10, or 40 Gy after 96 h. Cells were harvested, counted, and stained with AO/PI viability dye. Cell viabilities for each treatment, normalized to that of the mock-irradiated samples, are italicized. Data are representative of 2–3 independent experiments.

Overall, we found that the number of cells harvested did not significantly change after exposure to 2 and 4 Gy of ^223^Ra (Figure 1). The greatest effect on cell proliferation in the MDA-MB-231, LNCaP, PC3, H441, and H1703 tumor-cell lines was observed after the radiation dose was increased from 4 to 10 Gy. Surprisingly, only minor changes were observed in the total cell number when the amount of radiation was increased to 40 Gy of ^223^Ra. Cell viabilities (italicized in Figure 1) following each treatment were normalized to that of their respective mock-irradiated (0 Gy) controls. Cell viability was not less than 96% following 4 Gy of ^223^Ra in any of the tumor-cell lines; viability was at least 86% after 10 Gy of ^223^Ra in 5/6 cell lines. H441 had a viability of 76% after 10 Gy of alpha radiation. As was the case with the total cell number, viability underwent only minor changes when the dose was increased to 40 Gy. Unlike the other tumor cell lines, ZR75-1 was largely unaffected by ^223^Ra, as demonstrated by the slight reduction in proliferation rate and the lack of change in viability. Although the relative biological effectiveness values of ^223^Ra and EBRT are 20 and 1, respectively, both treatments had comparable effects on tumor-cell proliferation and viability [15].

### Exposure of human carcinoma cells to sublethal doses of ^223^Ra significantly increased sensitivity to antigen-specific CTL lysis

**Figure 2:**
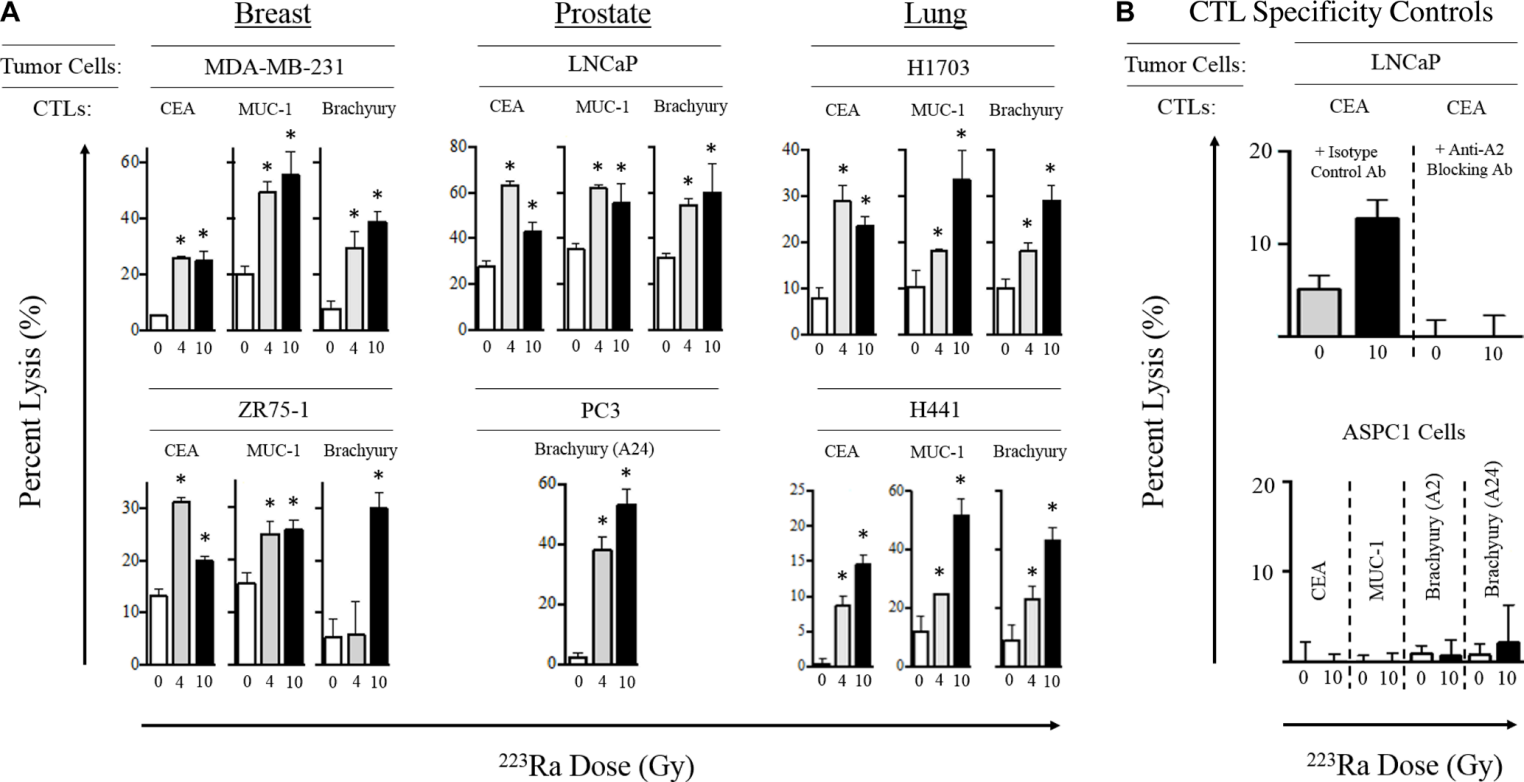
Sublethal exposure to ^223^Ra increases CTL lysis of breast, prostate, and lung carcinoma cells *in vitro* (**A)** Tumor cells were either left untreated (0 Gy, white bars) or exposed to 4 Gy (grey bars) or 10 Gy (black bars) of ^223^Ra over a 96-h incubation period, then used as targets in an overnight CTL lysis assay. CEA-, MUC-1-, and brachyury-specific CD8^+^ T cells were used as effectors at an E:T ratio of 30:1. (**B)** CTL HLA restriction was verified by incubating tumor cells with anti-HLA-A2 blocking mAb (top) or by performing the killing assay with CEA^+^HLA-A2^−^ AsPC-1 cells as target controls (bottom). Experiments were repeated 1–3 times with similar results. *denotes statistical significance relative to untreated cells (*P* < 0.05).

After establishing that ^223^Ra doses up to 40 Gy did not induce substantial tumor-cell death, we next examined whether alpha radiation affected the susceptibility of surviving tumor cells to CTL-mediated lysis. For this analysis, we focused primarily on the clinically relevant 4- and 10-Gy doses of ^223^Ra. Breast, prostate, and lung carcinoma cells were first exposed to ^223^Ra for 96 hours, then co-cultured with CD8^+^ effector T cells specific for carcinoembryonic antigen (CEA; HLA-A2-restricted), mucin-1 (MUC-1; HLA-A2-restricted), and brachyury (HLA-A2/A24-restricted) epitopes. As shown in Figure 2A, treatment with either dose of ^223^Ra increased the sensitivity of the breast, prostate, and lung carcinoma cell lines to CTL-mediated lysis targeting the CEA, MUC-1, and brachyury tumor antigens. We observed a significant increase in T cell-mediated killing of 2/2 breast, 1/1 prostate, and 2/2 lung tumor cell lines by CEA- and MUC-1-specific CTLs after both 4 and 10 Gy of radiation. However, no consistent dose-dependent effects were observed, as only 1/5 and 2/5 cell lines were killed to a greater degree with the CEA- and MUC-1-specific CTLs, respectively, at the higher dose of ^223^Ra. We also observed a significant increase in T cell-mediated killing of 1/2 breast, 2/2 prostate, and 2/2 lung carcinoma cell lines by brachyury-specific CTLs in response to 4 Gy of ^223^Ra. An increase in tumor-cell lysis occurred in all 6 cell lines following 10 Gy of radiation. Only 3/6 cell lines were more sensitive to brachyury-specific CTL-mediated lysis when the ^223^Ra dose was increased from 4 to 10 Gy. Interestingly, only the lung carcinoma cell lines (H1703 and H441) demonstrated a propensity to be killed at a greater level by each of the 3 antigen-specific CTLs at the higher dose of ^223^Ra.

We conducted 2 different CTL specificity control experiments to verify that the CTL killing was major histocompatibility complex class I (MHC-I)-restricted. The addition of an anti-HLA-A2 blocking antibody during the killing assay abolished CEA-specific CTL-mediated lysis of LNCaP tumor cells (HLA-A2^+^) exposed to 10 Gy ^223^Ra (Figure 2B). Moreover, exposure of the HLA-A2/A24^−^ AsPC-1 carcinoma cell line to 10 Gy of ^223^Ra over 96 hours did not result in any significant lysis by any of the CEA, MUC-1, or brachyury CTLs used in this study (Figure 2B, bottom panel). Taken together, these results indicate that sublethal doses of ^223^Ra enhance HLA-restricted, antigen-specific, CTL-mediated lysis of various human carcinomas, and that such killing can be achieved targeting a broad repertoire of tumor-associated antigens.

### Exposure of human carcinoma cells to ^223^Ra significantly increased expression of histocompatibility leukocyte antigens (HLA)

**Figure 3:**
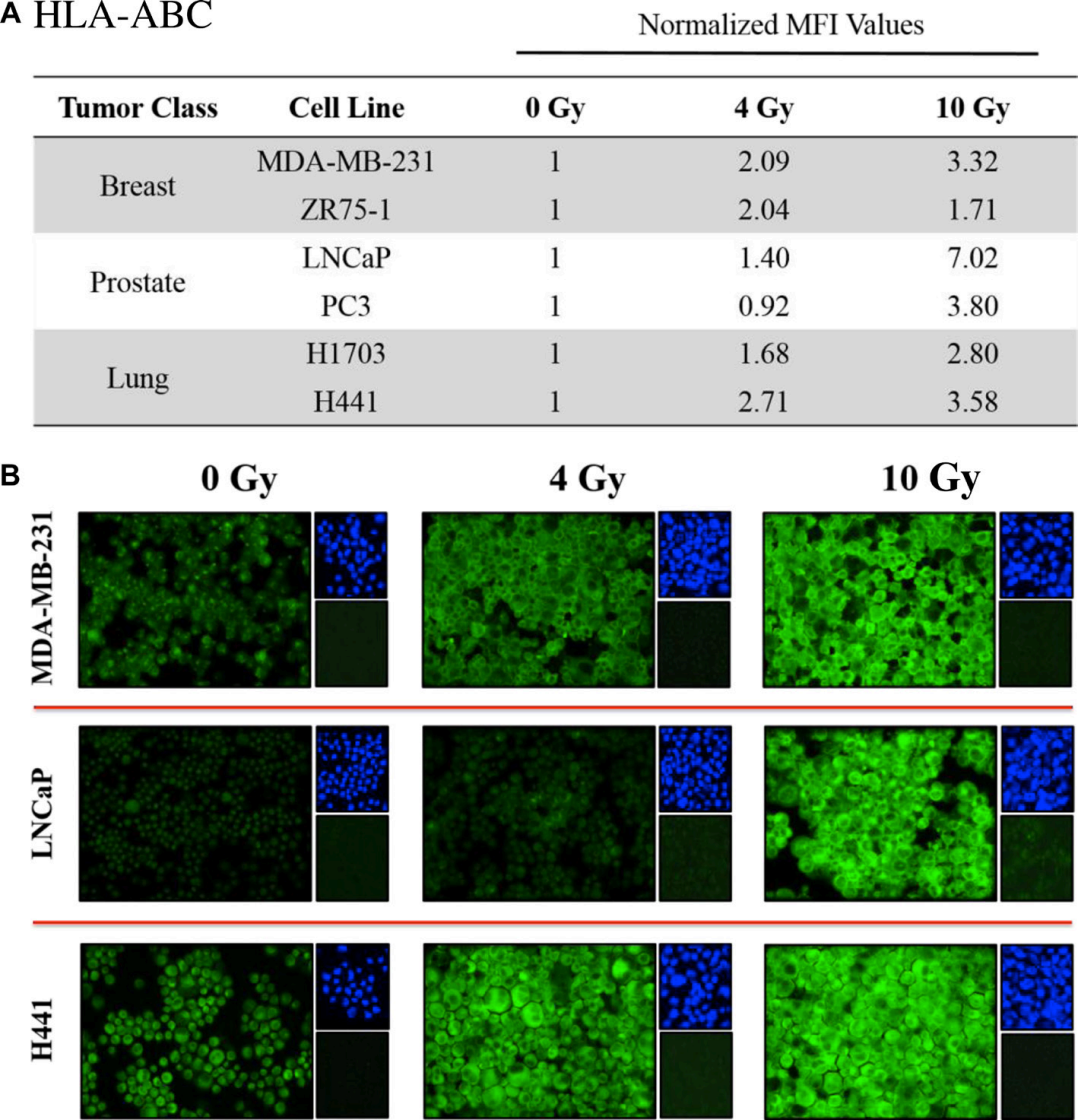
Sublethal doses of ^223^Ra significantly increase HLA expression in various tumor types Human prostate, breast, and lung carcinoma cells were mock-irradiated (0 Gy) or exposed to a cumulative dose of ^223^Ra totaling 4 or 10 Gy over a 96-h incubation period. Changes in HLA-ABC expression were determined by immunofluorescence imaging post-irradiation. (**A)** HLA-ABC expression was quantified using ImageJ software. The data presented are mean fluorescence intensity (MFI) values normalized to their respective mock-irradiated controls. (**B)** HLA-ABC immunofluorescence stains (green, 20× magnification). Upper right panels: 4′6-diamidino-2-phenylindole nuclear stain (blue). Lower right panels: isotype control. Data are representative of 2–3 independent experiments.

CTL killing requires interaction between the T-cell receptor and CD8-restricted epitopes presented by MHC-I molecules on the surface of tumor cells. We examined changes in HLA-A, B, and C (HLA-ABC) expression by immunofluorescence in each cell line following a 96-hour exposure to 4 and 10 Gy of ^223^Ra. A quantitative image analysis (Figure 3A) showed at least 2-fold increases in HLA-ABC for each cell line at either the 4- or 10-Gy dose relative to mock-irradiated controls. ^223^Ra induced dose-dependent upregulation of HLA-ABC in all prostate and lung tumor cell lines and in the MDA-MB-231 breast tumor cell line. At 10 Gy ^223^Ra, HLA-ABC expression increased 7.02-fold and 3.80-fold in LNCaP and PC3 prostate carcinoma lines, respectively. HLA-ABC also increased 3.32-fold and 1.71-fold in MDA-MB-231 and ZR-75-1 breast carcinoma cells, respectively, and 3.58-fold and 2.80-fold in H441 and H1703 lung tumor cell lines, respectively. Figure 3B presents a series of representative HLA-ABC immunofluorescence images at 0, 4, and 10 Gy ^223^Ra for each tumor type.

### Exposure of human carcinoma cells to ^223^Ra significantly increased expression of calreticulin

**Figure 4:**
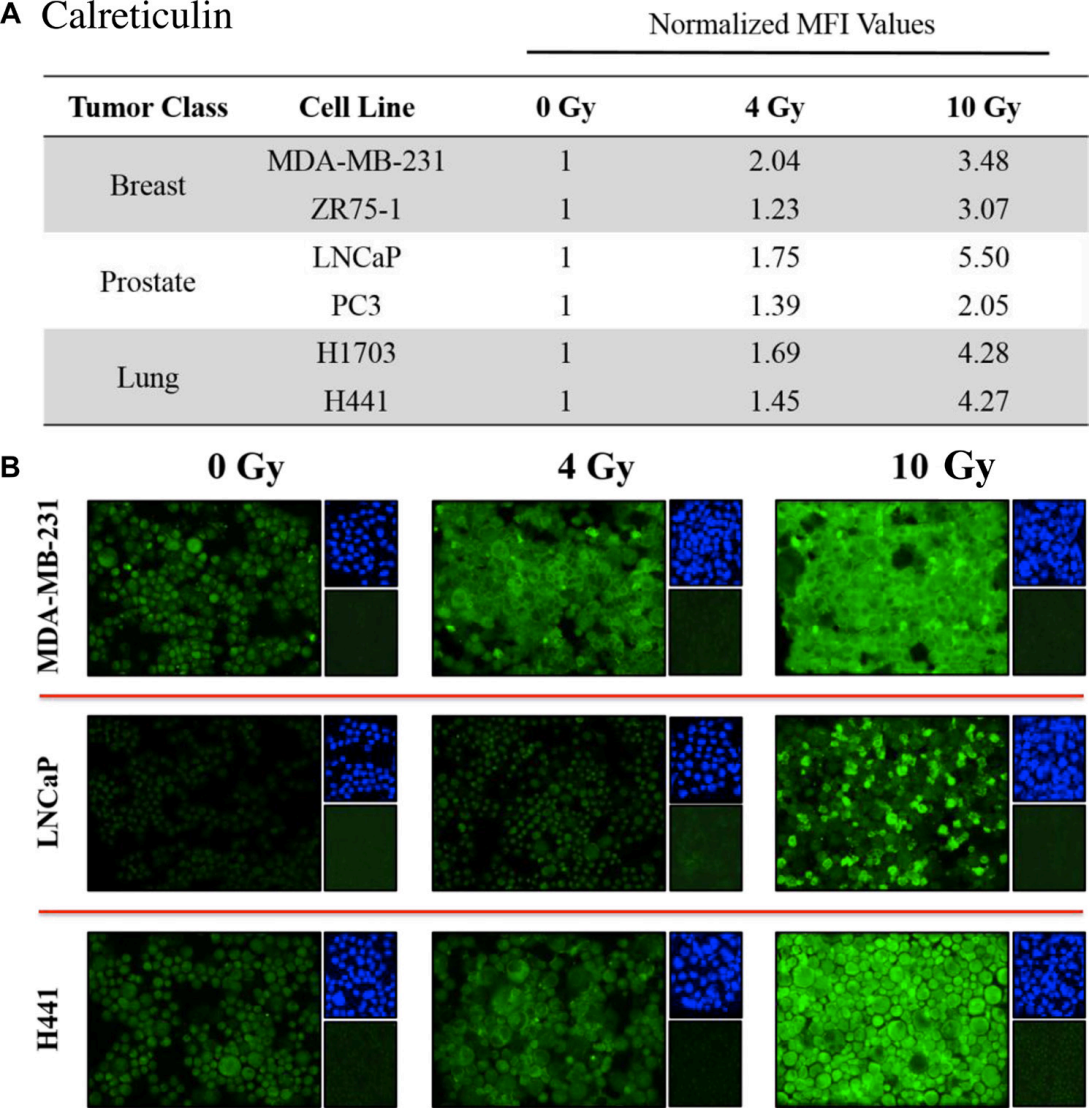
Tumor cells exposed to ^223^Ra have increased expression of calreticulin Immunofluorescence imaging was used to analyze calreticulin upregulation in human prostate, breast, and lung cancer cells following exposure to ^223^Ra. (**A)** Calreticulin expression was quantified using ImageJ software. The data presented are MFI values normalized to their respective mock-irradiated controls. (**B)** Calreticulin immunofluorescence stains following a cumulative dose of either 4 or 10 Gy radiation over a 96-h treatment period (green, 20× magnification). Upper right panels: 4′6-diamidino-2-phenylindole nuclear stain (blue). Lower right panels: isotype control. Data are representative of 2–3 independent experiments.

Calreticulin is a chaperone protein of the antigen-processing machinery (APM) that helps guide peptide loading into MHC-I molecules. Due to its potential role in modulating CTL sensitivity, we studied the effects of ^223^Ra on calreticulin expression by immunofluorescence. Similar to the results obtained for HLA-ABC, calreticulin was markedly upregulated upon exposure to ^223^Ra. We observed elevated levels of total calreticulin protein in all of the tested cell lines in response to 4 Gy of ^223^Ra, and the amount of calreticulin further increased after exposure to 10 Gy (Figure 4A). At 10 Gy ^223^Ra, calreticulin expression increased 5.5-fold and 2.5-fold in LNCaP and PC3 prostate carcinoma cells, respectively, compared to untreated controls. Calreticulin also increased 3.48-fold and 3.07-fold in MDA-MB-231 and ZR-75-1 breast carcinoma cells, respectively, and 4.27-fold and 4.28-fold in the lung tumor cell lines, respectively (Figure 4B).

### ^223^Ra induced the ER stress response and surface translocation of calreticulin in tumor cells

**Figure 5:**
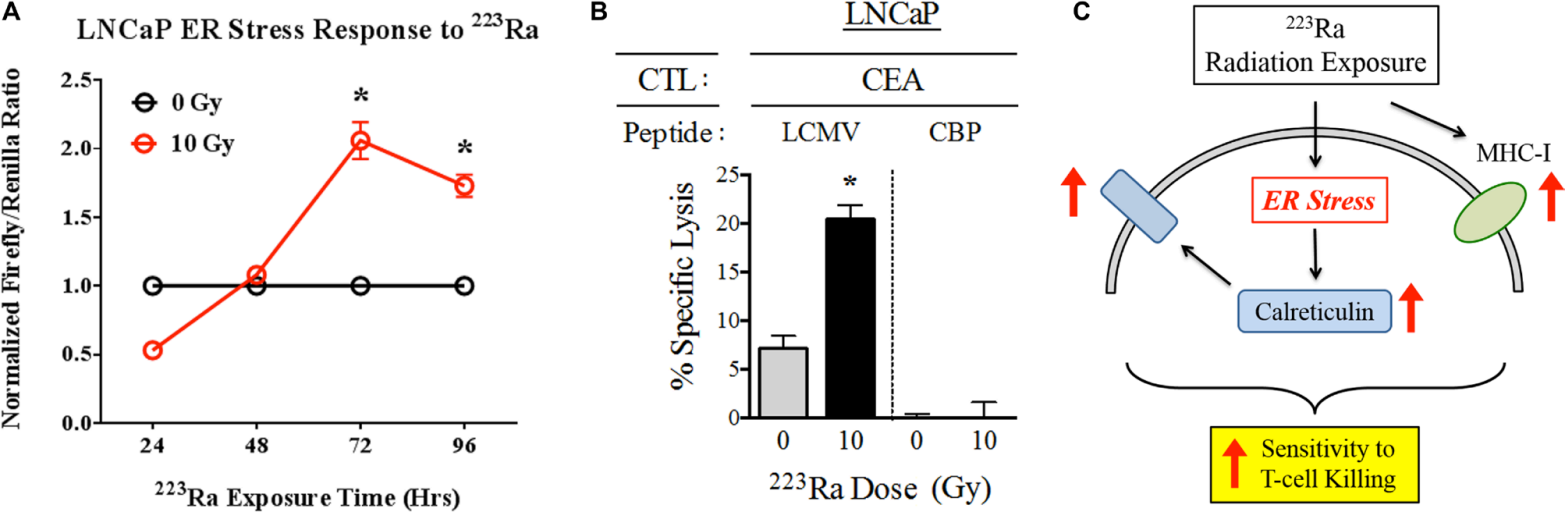
^223^Ra activates the ER stress response in LNCaP cells (**A)** LNCaP cells stably transduced with a firefly luciferase ER stress reporter element were either mock-irradiated (0 Gy) or exposed to a total dose of 10 Gy ^223^Ra over a 96-h incubation period. At the indicated time points, firefly and Renilla luciferase activities were determined using a Dual Luciferase Reporter Assay System. The results are shown as the ratio of firefly luciferase activity to Renilla luciferase control. (**B)** Functional role of cell-surface calreticulin on CTL-mediated lysis. LNCaP cells were either mock-irradiated or exposed to 10 Gy ^223^Ra, then co-cultured with CEA-specific CD8^+^ T cells in the presence of calreticulin blocking peptide (CBP, right) or lymphocytic chriomeningitis virus control peptide (LCMV, left). (**C)** Schematic illustrating the immunomodulatory effects of ^223^Ra on tumor cells. *denotes statistical significance relative to untreated cells (*P* < 0.05).

Radiation-induced immunogenic modulation of calreticulin in carcinoma cells is mediated by the endoplasmic reticulum (ER) stress response, as previously reported [25]. Based on the observed effects of ^223^Ra on calreticulin protein expression, we next examined whether this alpha emitter is similarly capable of activating the ER stress response in tumors. We stably transduced LNCaP prostate tumor cells with an ER stress response element that drives firefly luciferase expression, and then either mock-irradiated the cells or treated them with a cumulative dose of 10 Gy ^223^Ra over 96 hours. ^223^Ra triggered the ER stress response by 72 hours (*P* < 0.001) after the start of treatment, and the cells maintained this activated state at 96 hours (*P* < 0.001), at which time we performed CTL killing assays (Figure 5A).

One of the functional consequences of ER stress induction in tumor cells is translocation of calreticulin to the cell surface, where it enhances target killing by enhancing tumor-cell/T-cell recognition. We analyzed this immunomodulatory effect by investigating how an exogenous calreticulin blocking peptide, designed to inhibit the interaction between calreticulin and its receptor on T cells, would affect CTL killing following ^223^Ra exposure. As expected, 10 Gy of ^223^Ra induced greater CEA-specific CTL killing of LNCaP cells in the presence of a control lymphocytic choriomeningitis virus peptide LCM. However, the increase in CTL-mediated lysis was completely abrogated by the addition of calreticulin blocking peptide, demonstrating the importance of surface calreticulin in T-cell killing of tumor cells upon exposure to ^223^Ra. This result led us to develop a working model (Figure 5C) of immunogenic modulation of tumor cells following ^223^Ra therapy. Here, we show that while ^223^Ra increases expression of MHC-I, it can also induce ER stress in tumor cells and subsequently lead to upregulation and surface translocation of calreticulin. These phenotypic changes ultimately enhance tumor sensitivity to CTL- mediated lysis.

## DISCUSSION

The primary goal of radionuclide therapy is to reduce tumor burden through direct killing of tumor cells. Unfortunately, dose delivery constraints designed to limit toxicity allow for the survival of tumor cells that are not exposed to a lethal dose of radiation. Radiolabeled antibodies also are likely to be delivered in non-lytic doses because poor vascularization of solid tumor tissue hampers the infiltration of these agents [13]. However, radiotherapies delivered in sublethal doses can still be therapeutic anticancer modalities if they can take advantage of other immune-activating agents as part of a combination regimen [26, 27]. This may be achieved by inducing a spectrum of phenotypic changes in the surviving tumor cells that collectively augment their susceptibility to antigen-specific T cell-mediated destruction, a process known as immunogenic modulation. The changes that occur primarily involve multiple components of the APM, such as immunoproteosome subunits, peptide transporters, and protein chaperones, all of which contribute to enhanced presentation of tumor antigens for CTL recognition [19, 20, 25, 28].

^223^Ra is currently prescribed for patients with mCRPC at a dose of 55 kBq/kg of body weight (or 48 μCi/kg) [16]. For a 70-kg patient, assuming an absorbed radiation dose of 4.262 μCi/rad to osteogenic cells, this translates to approximately 4 Gy of ^223^Ra. In our studies, we exposed a variety of cell lines representative of tumors that frequently metastasize to bone, notably prostate, breast, and lung (Table 1), to ^223^Ra over 96 hours. Although cumulative doses of 4 and 10 Gy were found to be sublethal (Figure 1), they were still able to enhance T cell-mediated lysis of each tumor cell line by CTLs specific for MUC-1, brachyury, and CEA tumor antigens (Figure 2). The dose effect on CTL killing (Figure 2) did not entirely reflect the dose effect on surface MHC-1 (Figure 3). We have previously noted that radiation has several effects on antigen processing machinery independent of MHC-1 surface expression [20]. Doses as low as 2 Gy have been shown to increase tumor sensitivity to CTL killing. It would be challenging to target a given tumor exposure dose with ^223^Ra due to its uptake being dependent on osteogenic bone turnover rate. Given these observations, treating patients with the approved dose of ^223^Ra would be predicted to modulate tumor phenotype, resulting in enhanced T-cell killing.

In addition to greater CTL sensitivity, each tumor-cell line displayed some of the cardinal signs of immunogenic modulation following ^223^Ra therapy [20], such as upregulation of HLA-ABC (Figure 3) and calreticulin (Figure 4), both of which are important for effective antigen presentation. T cell-mediated killing relies on recognition of specific CD8-restricted epitopes associated with MHC-I molecules on the surface of tumor cells. However, proper loading of MHC-I molecules is guided in part by calreticulin, a critical chaperone protein that resides in the ER membrane. Calreticulin is a component of the APM essential for efficient antigen presentation, folding of newly synthesized glycoproteins, and enrichment of endogenous peptides in the ER. Increased calreticulin expression has been observed in various tumor-cell lines following *in vitro* and *in vivo* treatment with sublethal EBRT and proton radiation, and is believed to play a critical role in enhancing antigen-specific CTL recognition of tumor cells [22, 25].

Previous studies with EBRT and proton radiation therapy have shown that translocation of calreticulin to the cell surface is as important as its upregulation during immunogenic modulation [22]. Surface calreticulin has the ability to bind CTLs and enhance tumor-cell lysis, possibly due to more stable and/or prolonged tumor cell/T cell interaction. In previous studies, inhibiting this interaction through the use of a calreticulin blocking peptide significantly reduced the sensitivity of irradiated tumor cells to CTL-mediated lysis [18, 22]. Here, we observed the same effect after treatment with ^223^Ra (Figure 5). The presence of calreticulin blocking peptide during the CTL killing assay completely abrogated any increase in tumor-cell lysis that occurred following radiation treatment. This finding demonstrates that treatment with ^223^Ra induces calreticulin translocation, and that enhanced CTL killing is dependent on interaction with surface calreticulin.

Calreticulin surface exposure and ER stress have previously been linked in immunogenic modulation studies of EBRT, which led us to investigate whether ^223^Ra also induces ER stress in tumor cells. In response to EBRT, ER stress initiates the unfolded protein response, a protective cellular mechanism that attempts to restore ER homeostasis by arresting protein translation and upregulating multiple APM components and other immune-related proteins [25]. One of the immunologically relevant changes that occur during the ER stress response is calreticulin surface expression [20, 25]. Using an LNCaP cell line transduced with an ER stress reporter element, we discovered that ER stress is induced within the first 72 hours after ^223^Ra treatment (Figure 5). It is likely that ER stress is the main driver of calreticulin translocation and is thus responsible for increased tumor sensitivity to CTL lysis. However, future mechanistic studies incorporating the ER stress inducer thapsigargin will provide better insight into ER stress’s role in the immunomodulatory effects of ^223^Ra [25].

^223^Ra’s ability to drive immunogenic modulation and enhance CTL lysis in a variety of tumor-cell lines suggests that it has broad applicability in cancer therapy. Here, we show that ^223^Ra has the potential to treat a variety of human carcinomas of distinct origin and genotype. Sensitivity to CTL lysis was enhanced in prostate, breast, and lung tumor cells regardless of their p53, triple-negative, or K-Ras mutational status, respectively (Table 1), suggesting that ^223^Ra may be used to effectively treat bone metastases arising from each of these tumor types. Moreover, with the development of novel radionuclide-labeled antibodies, alpha particle-emitting agents such as ^223^Ra may be targeted to primary and metastatic tumor lesions not only in bone, but also in soft tissue sites throughout the body that generally exhibit poor uptake of bone-seeking radionuclides [15, 29–31].

Various studies are currently exploring the use of ^223^Ra in combination therapies, primarily for the treatment of mCRPC. ^223^Ra is under clinical evaluation in combination with immunomodulatory chemotherapy drugs (docetaxel), androgen-deprivation therapies (enzalutamide and abiraterone), and osteoclast-targeted agents, including bisphosphonates (zoledronic acid) and anti-RANKL antibodies (denosumab) [2, 4, 18, 32, 33]. The studies presented here support the use of therapeutic cancer vaccines that can generate antigen-specific T-cell responses and exploit the immunostimulatory environment created by ^223^Ra radiotherapy to achieve robust and effective antitumor responses [34–36]. Further studies are required to determine optimal timing for the use of ^223^Ra in combination with immunotherapies.

Such an approach with alpha-emitting ^223^Ra is based on preclinical studies demonstrating that beta-emitting ^153^Sm is able to induce immunogenic modulation and render tumor cells more amenable to immune-mediated killing [37]. A recent phase II study by our group demonstrated the clinical benefit of combining PSA-TRICOM, a poxviral-based cancer vaccine, with ^153^Sm in late-stage mCRPC [38]. PSA-TRICOM is designed to activate PSA-specific T cells and slow tumor growth by expanding the antigen repertoire of T cells through epitope spreading. The combination of ^153^Sm and PSA-TRICOM increased progression-free survival, enhanced the number of CD4^+^ and CD8^+^ PSA-specific T cells expressing type I cytokines, and decreased PSA and soluble CD40L levels in patient sera compared with ^153^Sm alone [38]. Our studies with both ^153^Sm and ^223^Ra show that, compared to radiotherapy with beta-emitters, radiotherapy with alpha-emitters safely delivers much higher energy to tumor tissue in a more localized manner, providing a rationale for investigating the combination of ^223^Ra and PSA-TRICOM for the treatment of mCRPC.

## MATERIALS AND METHODS

### Tumor-cell lines

Cell lines used in these studies were from tumors capable of metastasizing to bone. Cells of human breast (MDA-MB-231 and ZR-75-1), prostate (LNCaP and PC3), pancreatic (AsPC-1), and lung carcinomas (NCI-H1703 and NCI-H441) were obtained from American Type Culture Collection (Manassas, VA). Cells were maintained at 37°C/5% CO_2_ in cell culture medium according to the supplier’s specifications, supplemented with 10% FBS, 100 μg/mL streptomycin, and 100 units/mL penicillin.

### Tumor irradiation

Tumor cells were treated with the alpha particle-emitting radiopharmaceutical radium-223 dichloride (Xofigo^®^; ^223^Ra), obtained from Bayer Pharmaceuticals (Whippany, NJ) under a Material Cooperative Research and Development Agreement. Each cell line was cultured in normal growth medium supplemented with a volume of ^223^Ra that would deliver cumulative radiation doses totaling 2, 4, 10, or 40 Gy by the end of a 96-h treatment period. The amount of ^223^Ra added to each flask was mathematically derived by assuming a cell culture mass of 10 g and by applying the ^223^Ra dose constant of 4.2 × 10^−12^ Gy kg/Bq/sec [14]. After the 96-h incubation period, the irradiated culture medium was removed from the flasks and cells were harvested for subsequent analysis. Tumor cells in suspension were also irradiated with a single 10-Gy dose of EBRT (photons, Cs-137 source, Gammacell-40; AECL/Nordion, Ottawa, ONT) and then returned to culture for an additional 96 h.

### CD8^+^ CTLs

CTLs specific for CEA, MUC-1, and brachyury were used to study the susceptibility of cancer cells to CD8^+^ T cell-mediated killing following ^223^Ra irradiation. The CEA-specific CTLs were HLA-A2-restricted and recognized the epitope YLSGANLNL (CAP-1) [39, 40]. The MUC-1-specific CTLs were HLA-A2-restricted and recognized the epitope ALWGQDVTSV [41, 42]. The brachyury-specific CTLs were either HLA-A2- or HLA-A24-restricted and recognized the epitopes WLLPGTSTL (T-p2A) and KYQNEEITAL, respectively [43].

### Cytotoxicity assays

Tumor cells were either mock-irradiated (0 Gy) or exposed to 4 or 10 Gy of ^223^Ra for 96 h. After treatment, cells were trypsinized and used as targets in a standard CD8^+^ T-cell cytotoxicity assay. Tumor cells were metabolically labeled with ^111^In-oxyquinoline (Medi-Physics, Arlington Heights, IL) and then co-incubated in 96-well round-bottom plates overnight at 37°C/5%CO_2_ with the CEA, MUC-1, and brachyury HLA-restricted CTL cell lines at an effector:target ratio of 30:1. Supernatant fluids were harvested and measured on a gamma counter to quantify the amount of ^111^In release. CTL killing was calculated according to the following formula: % specific lysis = (experimental release – spontaneous release) / (maximum release – spontaneous release) × 100. Spontaneous and maximum releases were determined by incubating the tumor cells with either regular culture medium or upon lysis with 2% Triton X-100 (Sigma-Aldrich, St. Louis, MO), respectively. CTL specificity control experiments were performed in the presence of anti-HLA-A2 or isotype control IgG2b antibodies (Bio-Rad Laboratories, Hercules, CA) at a concentration of 20 μg/mL. Negative control cytotoxicity assays were also performed with the AsPC-1 cell line (HLA-A2^−^, HLA-A24^−^/CEA^+^, brachyury^+^, MUC-1^+^) to confirm HLA specificity. For indicated experiments, the CTL assay was performed in the presence of calreticulin blocking peptide (MBL International, Woburn, MA) or lymphocytic choriomeningitis virus peptide NP_118−132_ (CPC Scientific, Sunnyvale, CA).

### Immunofluorescence

After a 96-h exposure to ^223^Ra, expression of calreticulin and HLA-ABC were analyzed by immunofluorescence. A total of 1 × 10^5^ cells were deposited onto microscope slides using cytocentrifugation (Shandon Cytospin; Thermo Fisher Scientific, Waltham, MA), and then fixed with 3% methanol-free paraformaldehyde (Electron Microscopy Sciences, Hatfield, PA) for 20 min. After permeabilization with 0.05% Triton X-100, the tumor cells were stained with mouse monoclonal antibodies specific for calreticulin (Abcam, Cambridge, UK) or HLA-ABC (BD Biosciences, San Jose, CA) at a 1:100 dilution, followed by an anti-mouse Alexa Fluor-488 conjugated secondary antibody (Thermo Fisher Scientific). The cells were then counterstained with 4′6-diamidino-2-phenylindole and examined by fluorescence microscopy (Leica Microsystems, Wetzlar, Germany). Relative calreticulin and HLA-ABC expression levels were calculated using ImageJ software by normalizing the intensity values to their respective mock-irradiated (0 Gy) controls.

### Luciferase ER stress reporter assay

LNCaP prostate tumor cells were stably transduced to express firefly luciferase under control of the ER stress transcriptional response element, as previously described [44]. As an internal control, these cells were also transduced to constitutively express Renilla luciferase (Thermo Fisher Scientific) under control of the cytomegalovirus promoter. Transduced cells were selected in medium containing 1 μg/mL puromycin (Life Technologies, Carlsbad, CA), and single-cell clones were either mock-irradiated (0 Gy) or exposed to 10 Gy ^223^Ra over a 96-h incubation period. Luciferase activity was quantified using the Dual-Luciferase Reporter Assay System (Promega, Madison, WI) at multiple time points after the start of treatment to monitor the progressive onset of the ER stress response pathway.

### Statistical analysis

Statistical analyses were performed using 2-tailed Student’s *t*-tests with a 95% confidence interval. All analyses were conducted using Prism Graphpad 6.0f software, and probability values of *P* < 0.05 were considered significant.
